# Evaluation of the Levetiracetam treatment on reduction of epileptic discharges in electroencephalogram in children with epilepsy

**DOI:** 10.22037/ijcn.v16i1.30946

**Published:** 2021

**Authors:** Afshin FAYYAZI, Mohammad Hossein EBRAHIMI, Ghodratollah ROSHANAEI, Hassan BAZMAMOUN

**Affiliations:** 1 Department of pediatric neurology, Hamadan University of Medical Sciences, Hamadan, Iran.; 2 Pediatrician, department of pediatrics, Hamadan University of Medical Sciences, Hamadan, Iran.; 3 Department of Biostatistics and Epidemiology, School of Public Health, Hamadan University of Medical Sciences, Hamadan, Iran.; 4 Department of pediatric gastroenterology, Hamadan University of Medical Sciences, Hamadan, Iran.

**Keywords:** Epilepsy, Levetiracetam, Sodium Valproate, Epileptic Discharges, Interictal Phase

## Abstract

**Objectives:**

Epilepsy is a relatively common disease in childhood. In some patients, the electroencephalogram (EEG) is abnormal despite the clinical control of seizures. Studies have identified the adverse effects of epileptic discharges on cognition, learning, behavior, and seizure recurrence in children. This study investigated the Levetiracetam effect on epileptic discharges in the interictal phase of EEG in epileptic children.

**Materials & Methods:**

This clinical trial was conducted on 54 epileptic children aged 2 to 15 years, whose clinical seizures were controlled by sodium valproate in the last six months but still had epileptic discharges in EEG. The participants were divided into two groups: an intervention group (21 people), for which Levetiracetam was added to sodium valproate, and a control group (33 people), for which treatment with sodium valproate alone was continued. The patients were then followed for one year.

**Results:**

The percent normalization of epileptic discharges in the intervention and control groups was 66.7% and 57.1% (P = 0.500), respectively. Also, the mean time for the normalization of epileptic discharges in the intervention and control groups was 12.60±8.25 and 20.57±12.67 months (*P* = 0.042), respectively.

**Conclusion:**

In children with controlled seizures whose EEG was still abnormal, sodium valproate therapy alone and combined with Levetiracetam effectively reduced the severity of epileptic discharges. However, the addition of Levetiracetam to sodium valproate normalized EEG more rapidly.

## Introduction

Epilepsy is the most common chronic neurological disorder in children, with a prevalence of about 1% on average (1). The epilepsy prevalence is reported to be 3.2-5.5 per 1000 in developed countries and 3.6-44 per 1000 in underdeveloped countries (2). In some of the epileptic children, EEG shows epileptic discharges despite the clinical control of seizure.

Studies have shown that epileptic discharges in children, even in the absence of clinical seizures, can be associated with cognitive, verbal, memory, and behavioral disorders (3-5). Epileptic discharges have been reported in 24% of children with ADHD (6). The clinical recurrence of seizures is more likely in people with abnormal EEG than in those with normal EEG (7). Sodium valproate is a widely used anti-epileptic drug with broad-spectrum activity that has been the first-line drug for most seizure types in children (8). Levetiracetam is also one of the new anticonvulsant drugs used to control many types of pediatric seizures (9, 10). There are some pieces of evidence that Levetiracetam can reduce epileptic discharges in the interictal phase in epileptic patients (11-13). Kanemura et al. (2018), in their study, compared the efficacy of carbamazepine and valproate sodium with Levetiracetam in reducing rolandic discharges on interictal electroencephalogram in children with rolandic epilepsy. They showed that Levetiracetam was superior to carbamazepine and valproate sodium in suppressing discharges in children with rolandic epilepsy (11).

In a multicenter, prospective, long-term study, Speechio et al. (2008) evaluated the effects of Levetiracetam on EEG abnormalities and the photoparoxysmal response of patients with juvenile myoclonic epilepsy. Efficacy parameters were based on comparing and analyzing EEG interictal abnormalities classified as spikes and waves, polyspikes and waves, and the presence of PPR. Levetiracetam effectively decreased epileptiform EEG abnormalities and suppressed the photoparoxysmal response of patients with juvenile myoclonic epilepsy (13).

The use of Levetiracetam also reduces epileptic discharges in other non-seizure disorders, such as ADHD. This drug has fewer side effects and drug interactions than other first-line drugs (14, 15).

 As previously mentioned, epileptic discharges in EEG can cause impairment in cognitive and behavioral development and increase the risk of recurrence in children. Thus, this study aimed to investigate the decreasing effect of adding Levetiracetam to sodium valproate on epileptic discharges in the interictal phase in epileptic children who still have abnormal EEG despite the clinical control of seizures. 

## Materials and methods

This clinical trial was conducted on 54 epileptic children aged 2 to 15 years admitted to the department of pediatric neurology of the Besat Hospital, Hamadan, Iran, in 2018. Clinical seizures in the patients were controlled by sodium valproate in the last six months, but they still had epileptic discharges in EEG.

The patients were divided into two groups: an interventional group (21 people), for which Levetiracetam tablets (manufactured by Cobel Company with a dose of 50 mg/kg and blood levels above 5 mg/L) were added to sodium valproate (manufactured by Rahakin Company with a dose of 20-30 mg/kg and blood levels above 50 mg/L) and a control group (33 people), for which treatment with sodium valproate alone was continued.

Then, the patients were monitored for seizure recurrence, drug side effects, and EEG changes every three months, for a period of one year. Periodic CBC and liver function tests were performed in both groups.

The patients' demographic information was collected using a checklist.

The inclusion criteria were children aged 2 to 15 years with idiopathic generalized tonic, clonic, or tonic-clonic seizures and tonic or clonic idiopathic focal seizures, no clinical seizures in the last six months, Spike-Wave epileptic discharges in EEG, and sodium valproate consumption for at least six months with a 20-30 mg/kg dose and blood levels above 50 mg/dl.

The exclusion criteria included the need to change the treatment regimen and initiate other anticonvulsant drugs and patients not providing consent or cooperation to participate in the study.

In children with good cooperation, EEG was performed in awake children with eyes open and closed for 20 minutes with hyperventilation and optical stimulation.

In non-cooperative children, EEG was performed for 20 minutes under sedation using chloral hydrate syrup.

EEG was recorded using 10-20 methods with a 16-channel Ebeneuro device. A pediatric neurologist studied all ECGs and determined the severity of epileptic seizures.

The severity of epileptic discharges (according to the number of epileptic discharges) was divided into four groups.

1. Normal

2. Mild: Epileptic discharges in some EEG pages (each page contains 10 seconds of EEG recording)

3. Moderate: Epileptic discharges in all EEG pages but with a distribution of less than 20% per page

4. Severe: Epileptic discharges in all EEG pages with the distribution of epileptic discharges in more than 20% of EEG pages

After collecting the data, SPSS software version 21 was used to analyze the data. Fisher's exact test and the chi-square test were used to evaluate the association between the two categorized variables, and the Z-score test was used to compare normal percent changes between the groups after the intervention. Moreover, an independent t-test and the Mann-Whitney U test were used to compare means concerning normal and un-normal measurements distribution, respectively. The significance level of the tests was considered less than 0.05.

This study was conducted with the approval of the Ethics Committee of the Hamadan University of Medical Sciences with the ethics code, the Deputy of Research and Technology IR.USHA.REC.1397.146, IRCT20120215009014N246. **Informed consent** was obtained from all the patients or their parents.

## Results

There was no significant difference between the two groups regarding age, sex, the severity of seizures (Tables 1 and 2). EEG normalization became normal in 15 (71.4%) of patients in the intervention group and 21 (63.16%) of patients in the control group. However, the frequency of EEG normalization was higher in the intervention group than in the control group, although not statistically significant (P = 0.554).

In both groups, the severity of epileptic discharges significantly decreased after the treatment compared to before the treatment (P< 0.001). However, the severity of EEG abnormalities did not show a statistically significant difference (Table 2) between the two groups before and after the treatment. The recurrence of clinical seizures occurred in seven (21.2%) of patients in the control group and two (9.5%) of patients in the intervention group (P = 0.256).

The mean time for normalization of the first EEG was 12.60±8.25 and 20.57± 12.67 months (*P* = 0.042) in the intervention and control groups, respectively. Moreover, no significant drug side effects leading to discontinuation of the treatment were observed in either group.

**Table 1:** Distribution of demographic and clinical characteristics between two groups

‍Control group	Intervention group	Variable
Number(Percent)	Number(Percent)	Gender
(%63.6) 21 (%36.4) 12 (%100) 33	(%71.4) 15 (%28.6) 6 (%100) 21	Male Female Total
0.554		P.value
2.84±6.95	3.38±6.60	Age(yrs) Mean±Sd
0.672		P.value

**Table 2:** Severity of interictal epileptic discharges in EEG before and after treatment in two groups

p-value	‍‍‍‍‍‍‍‍Children with idiopathic epileptic seizures		Severity of discharges	
	Intervention group Number(Percent)	‍Control group Number(Percent)		
100	(%0) 0 (%95.2) 20 (% 4.8) 1 (%100) 21	(%3) 1 (%93.9) 31 (%3) 1 (%100) 33	Mild Moderate Severe Total	Before
0.430	(%71.4) 15 (%9.5) 2 (%14.3) 3 (%4.8) 1 (%100) 21	(%57.6) 19 (%27.3) 9 (%12.1) 4 (0/3) 1 (%100)	Normal Mild Moderate Severe Total	After

## Discussion

This study showed that the patients' EEG was normalized in a shorter period by adding Levetiracetam to sodium valproate. Moreover, the number of cases with normalized EEG was higher in the intervention group, although not significant. This may be due to the small number of people under study.

Our study showed that sodium valproate therapy alone and in combination with Levetiracetam effectively corrected epileptic discharges in EEG. However, the addition of Levetiracetam to sodium valproate did not significantly alter the effect of sodium valproate alone on EEG.

Previous studies have shown that sodium valproate is effective in correcting EEG (5, 8). Kanemura et al. and Wang et al., in their studies showed the Levetiracetam efficacy in correcting epileptic discharges in EEG (11,12), although Levetiracetam was not effective in Stodieck et al.’s study (16). 

Numerous studies have shown that epileptic discharges in children, even in the absence of clinical seizures, can be associated with cognitive, verbal, memory, and behavioral disorders (3). Previous studies have also shown that anticonvulsant drug therapy in children with developmental disorders that have abnormal EEG without clinical seizures improves the course of development (4, 5, 17). In Liu Z et al.'s study, Levetiracetam combined with sodium valproate produced better efficacy and fewer adverse reactions, significantly improved patients' quality of life and cognitive function, and notably lowered the content of neurological function indicators with notable EEG improvement (18).

Also, in children with behavioral disorders and ADHD who have abnormal EEG without clinical seizures, the correction of EEG has been effective in improving behavioral disorders (6).

Although in our study, adding Levetiracetam to sodium valproate did not significantly increase the extent of recovery, it shortened the meantime for EEG normalization by about eight months.

## In Conclusion

Adding Levetiracetam to sodium valproate in children with idiopathic epilepsy, who have abnormal EEG despite controlling clinical seizures, shortens the time to achieve normal EEG. 
